# Spontaneous hemorrhage from a functionally well-controlled, non-enlarged but hyperplastic parathyroid gland due to secondary hyperparathyroidism: a case report

**DOI:** 10.1007/s13730-025-01059-1

**Published:** 2026-01-21

**Authors:** Daisuke Honda, Isao Ohsawa, Fuminori Nomura, Ai Watanabe, Kohei Kajino, Hiromichi Gotoh, Katsuhiko Asanuma

**Affiliations:** 1 Department of Nephrology, Graduate School of Medicine, Chiba University, 1-8-1 Inohana, Chuo-ku, Chiba-shi, Chiba 260-8677 Japan; 2 Nephrology Unit, Internal Medicine, Saiyu Soka Hospital, Saitama, Japan; 3 Department of Otorhinolaryngology, Soka Municipal Hospital, Saitama, Japan; 4 Department of Head and Neck Surgery, Institute of Science Tokyo, Tokyo, Japan

**Keywords:** Chronic kidney disease, Deep cervical hematoma, Hyperplasia, Parathyroid gland hemorrhage, Parathyroid hormone, Secondary hyperparathyroidism

## Abstract

Chronic kidney disease causes secondary hyperparathyroidism (SHPT) with hyperplastic enlarged parathyroid glands. Deep cervical spontaneous hemorrhage has been occasionally reported to be caused by rupture of an enlarged parathyroid gland under functionally uncontrolled SHPT. We encountered an unusual case of life-threatening spontaneous hemorrhage from a parathyroid gland in a patient undergoing hemodialysis. A 40-year-old man with end-stage renal disease received renal replacement therapy for 12 years. He had SHPT, which was functionally well controlled using multiple medications. The parathyroid glands had not been reported to be enlarged. He presented with sudden-onset progressive cervical swelling and pain without any specific triggers. Computed tomography revealed hematoma formation around the thyroid gland and in the upper mediastinum, as well as a clearly defined high-density mass shadow on the right dorsal surface of the thyroid gland. Therefore, the patient was diagnosed with parathyroid gland hemorrhage. The next day, emergency surgery was performed because the hematoma had expanded and his clinical symptoms had worsened. Pathology revealed that the excised right lower parathyroid gland measured 7 mm, was non-enlarged, and comprised hyperplastic parathyroid tissue with hemorrhage. Our report indicates that severe parathyroid gland hemorrhage can occur even in functionally well-controlled SHPT with hyperplastic but non-enlarged parathyroid glands. Because spontaneous parathyroid gland hemorrhage may be linked to a relative imbalance between parathyroid gland cell growth and blood supply, regular evaluation of blood flow and the parathyroid glands using ultrasonography is important in patients with SHPT.

## Introduction

Chronic kidney disease (CKD) is associated with mineral and bone disorders that are serologically characterized by hypocalcemia and hyperphosphatemia. Decreased serum calcium or increased serum phosphorus levels activate the parathyroid glands, which progressively secrete parathyroid hormone (PTH) [1, 2]. This condition is known as secondary hyperparathyroidism (SHPT) and is characterized by pathological hyperplasia and noticeable enlargement of the parathyroid glands [3].

Functionally uncontrolled SHPT increases the risk of bone deformities and cardiovascular events caused by atherosclerosis [2]. Furthermore, in rare cases, rupture of an enlarged parathyroid gland could lead to life-threatening deep cervical spontaneous hemorrhage [4, 5]. Thus, an elevated PTH level is considered a useful biomarker for differentiating parathyroid gland hemorrhages from other causes of deep cervical hematomas [6]. Nagasawa et al. reported a case of functionally well-controlled SHPT with normal PTH levels in a patient who had parathyroid gland hemorrhage and underwent urgent life-saving parathyroidectomy [5]. However, the authors did not provide information on the size of the associated parathyroid gland, although an encapsulated mass was detected on computed tomography (CT) after the hemorrhage.

We encountered a case of severe deep cervical hematoma due to spontaneous hemorrhage from a hyperplastic parathyroid gland in a patient undergoing hemodialysis. The patient had SHPT but achieved a functionally well-controlled condition without noticeable enlargement of the parathyroid glands after several medications. Therefore, we report an uncommon case with novel findings and perspectives that differ from those of previous reports in this field.

## Case report

**Fig. 1:**
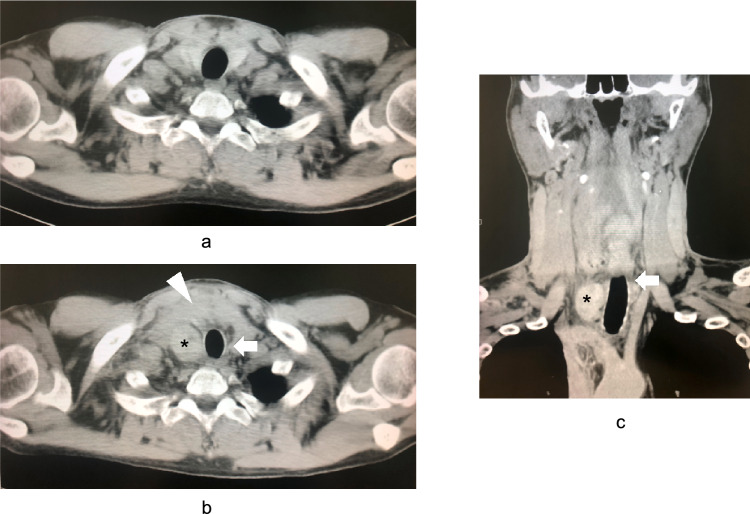
Computed tomography (CT) scan (**a**, **b**: axial section, **c**: coronal section). **a**: CT scan of the cervical region performed 9 days before the onset of cervical swelling showed no abnormal findings. **b**, **c**: Hematoma formation can be observed around the thyroid gland and in the upper mediastinum (white arrowhead), with a leftward shift and compression of the trachea (white arrow). A clearly defined high-density mass shadow can be observed on the right dorsal surface of the thyroid gland (black asterisk). Figure **b** and **c** were reproduced from Reference [7]

**Fig. 2:**
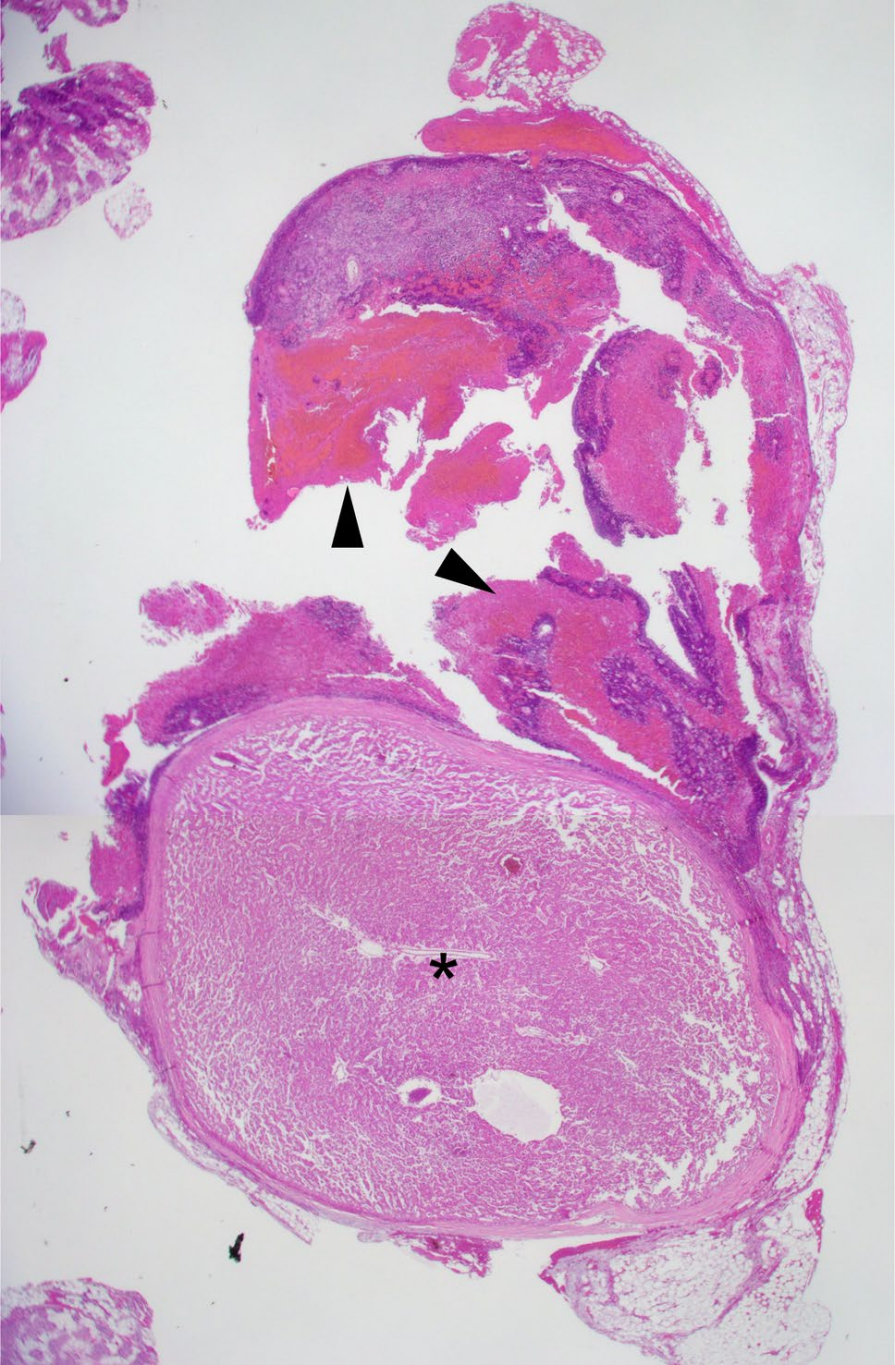
Resected right lower parathyroid gland. A 7-mm hyperplastic nodular lesion (black asterisk) can be observed with hemorrhage (black arrowheads). Figure 2 was reproduced from Reference [7]

**Fig. 3:**
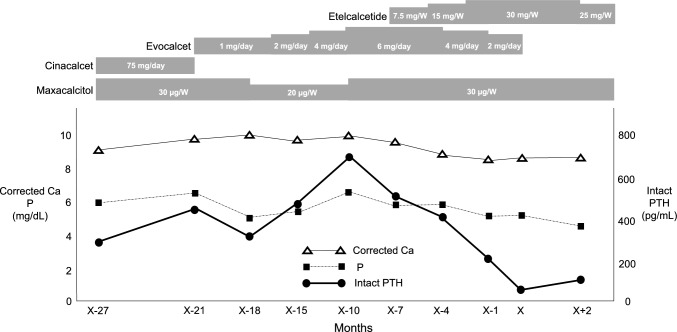
Clinical course of the patient with spontaneous parathyroid gland hemorrhage. X in the horizontal axis means indicates the time when the patient experienced the spontaneous parathyroid gland hemorrhage. Serum levels of intact PTH, corrected calcium, and phosphorus are shown. SHPT was treated with multiple medications, such as maxacalcitol, cinacalcet, evocalcet, and etelcalcetide. SHPT, secondary hyperparathyroidism; PTH, parathyroid hormone; Ca, calcium; P, phosphorus; W, week; corrected Ca (mg/dL) = calcium (mg/dL) + 0.8 × (4.0 − albumin (g/dL))

The patient was a 40-year-old man without any hematological disorders that could lead to hemorrhage. He was started on peritoneal dialysis for end-stage renal disease due to IgA nephropathy at the age of 28 years and switched to hemodialysis at the age of 30 years. He had SHPT as one of the typical complications related to CKD, which was relatively well controlled (intact PTH: 219.5 pg/mL, phosphorus: 5.5 mg/dL, and corrected calcium [= calcium (mg/dL) + 0.8 × (4.0 − albumin (g/dL)]: 8.5 mg/dL) with multiple medications (evocalcet 2 mg/day, etelcalcetide hydrochloride 10 mg/session, and maxacalcitol 10 µg/session). Although routine ultrasonography examinations were not performed, no parathyroid gland enlargement was noted on periodic CT examinations. At the age of 40 years, the patient presented with acute progressive anterior right cervical swelling, pain, and mild dysphagia without any specific triggers. His consciousness was clear, with a blood pressure of 146/95 mmHg, body temperature of 36.6 ℃, and oxygen saturation level of 99% in room air. The patient’s neck was swollen, and his respiratory symptoms persisted. His blood analysis showed no abnormal thyroid function or severe inflammation (thyroid-stimulating hormone: 0.967 µIU, free triiodothyronine: 1.91 pg/mL, free thyroxine: 0.93 ng/dL, white blood cell count: 8800/µL, C-reactive protein: 1.55 mg/dL). Because the patient had a history of bronchial asthma, plain CT was performed instead of enhanced CT. The plain CT revealed hematoma formation around the thyroid gland and in the upper mediastinum, causing a leftward shift of the trachea, and a clearly defined high-density mass shadow on the right dorsal surface of the thyroid gland, which was considered to be the source of the hemorrhage, although periodic CT scan of the cervical region performed 9 days before the onset of cervical swelling showed no abnormal findings (Fig. 1) [7]. Laryngoscopy revealed no obstruction of the respiratory tract, although there was evidence of submucosal hemorrhage. The patient was diagnosed with a deep neck cervical hematoma due to hemorrhage from the right lower parathyroid gland. At that time, the laboratory data showed intact PTH: 52.0 pg/mL, phosphorus: 5.4 mg/dL, and corrected calcium: 8.4 mg/dL. He was admitted for follow-up observation because of the possibility of symptom exacerbation that could lead to airway obstruction. He had severe cervical pain, for which he was administered pentazocine. The following day, his clinical symptoms worsened as the hematoma expanded and caused more tracheal compression, with a risk of fatal suffocation. Subsequently, emergency surgery was performed at the Department of Otorhinolaryngology. No active bleeding was observed macroscopically during the surgery. Although a parathyroid gland was located at the center of the hematoma, there was no obvious capsular rupture or cyst. The inferior thyroid artery and its branches were in close proximity to the parathyroid gland, but no signs of bleeding from the vessels were observed. The hematoma and right lower parathyroid gland were removed, and tracheocutaneous fistuloplasty was performed under general anesthesia. Pathologically, the excised right lower parathyroid gland measured 7 mm, and was non-enlarged, composed of hyperplastic parathyroid tissue, and associated with a nodular lesion, capsular rupture, and hemorrhage (Fig. 2) [7]. Therefore, extracapsular hemorrhages from the parathyroid gland were identified owing to the fragility of the parathyroid gland capsule. After surgery, his cervical swelling improved, and there was no recurrence of hemorrhage after heparin administration during hemodialysis sessions. The tracheocutaneous fistula was repaired surgically on the 8th postoperative day. He was discharged on the 13th postoperative day without obvious complications related to parathyroidectomy, such as recurrent laryngeal nerve palsy or hypocalcemia with a decreased PTH level (intact PTH: 81.6 pg/mL, phosphorus: 4.4 mg/dL, and corrected calcium: 8.8 mg/dL). The evocalcet treatment for SHPT was discontinued (Fig. 3).

## Discussion

The underlying conditions causing cervical swelling and pain include infections, abscesses, tumors, and deep cervical hemorrhage. In a few cases, deep cervical hemorrhage can originate from the parathyroid gland. As the parathyroid glands are normally small (≤ 8 mm) and located deep in the neck, they are not naturally damaged by external pressure. However, an enlarged parathyroid gland causes hemorrhage mainly by traumatic rupture of small blood vessels and infrequently by spontaneous rupture associated with functionally uncontrolled hyperparathyroidism because the parathyroid gland capsule is thin [3–6, 8]. We reviewed eight studies published since 2000 that reported definite information on the size of the resected parathyroid in cases of spontaneous rupture and cervical hemorrhage (Table 1). Seven of them reported the existence of primary hyperparathyroidism with adenoma and one noted a cyst, but no SHPT was observed. Each parathyroid gland that ruptured spontaneously was enlarged, and the patient underwent standby or urgent surgery for the hemorrhage, with good outcomes.

**Table 1:** Previous studies on spontaneous hemorrhage from a parathyroid gland

	Author (year)	Age (year)/Sex	Presentation	Parathyroid gland size (mm)	Histology/HPT type	Management	Intact PTH (pg/mL)	Ca (mg/dL)
1	Kihara et al. [6]	60/F	Extensive ecchymosis of the neck	28 × 17 × 10	Adenoma/primary	Standby surgery	120	11.1
2	Nakajima et al. [9]	70/M	Chest oppression and pain, and subcutaneous hemorrhage in the anterior neck	35 × 10 × 10	Adenoma/primary	Standby surgery	Not reported	9.7
3	Taniguchi et al. [10]	41/F	Neck swelling and pain, dysphagia, and dysphonia	15 × 15 × 60	Cyst	Standby surgery	Not reported	Not reported
4	Merante-Boschin et al. [11]	56/F	Painful, right-sided neck mass	40 × 24 × 13	Adenoma/primary	Emergency surgery	Not reported	12.7
5	Shinomiya et al. [12]	76/F	Pharyngeal discomfort and extensive ecchymosis over the neck and upper anterior chest wall	35 × 25 × 15	Adenoma/primary	Standby surgery	453	11.6
6	Shinomiya et al. [12]	62/M	Anterior chest wall ecchymosis	28 × 19 × 17	Adenoma/primary	Standby surgery	201	11.7
7	Khan et al. [13]	69/F	Sensation of globus, mild dysphagia, neck swelling, and bruising extending down to the chest	23 × 12 × 8	Adenoma/primary	Standby surgery	Not reported	10.7
8	Matías-García et al. [14]	78/F	Dyspnea during moderate exertion and odynophagia	60 × 40 × 30	Adenoma/primary	Emergency surgery	Not reported	Not reported

*HPT* hyperparathyroidism, *PTH* parathyroid hormone, *Ca* calcium, *F* female, *M* male

Although this patient did not have noticeable enlargement of the hyperplastic parathyroid glands (7 mm) or poorly controlled PTH levels, the patient experienced parathyroid gland hemorrhage. In such a situation, we can consider that the parathyroid gland hemorrhage was caused by a relative imbalance between parathyroid gland cell growth and blood supply, as described by Roma et al. [4]. Previous reports have suggested that treatment for SHPT might destabilize the balance between blood flow and the growth/apoptosis of parathyroid gland cells. Meola et al. reported that hypervascular parathyroid glands are hypovascular or avascular in response to a calcimimetic agent, and Mizobuchi et al. demonstrated that a calcimimetic agent can cause apoptosis in parathyroid gland cells [15, 16].

We conclude that it is important to recognize the possibility of life-threatening parathyroid gland hemorrhage, even in patients with functionally well-controlled and non-enlarged but hyperplastic parathyroid glands due to SHPT, among those with CKD who develop a deep cervical hematoma. Furthermore, we must reconsider the importance of regular morphological and hemodynamic evaluations of the parathyroid glands in addition to functional assessment in patients with SHPT.
